# Incidental synchronous bronchial tumour: an unusual bronchoscopic finding

**DOI:** 10.1002/rcr2.585

**Published:** 2020-05-17

**Authors:** Umberto Caterino, Dario Amore, Chiara Petagna, Albina Palma, Danila Caroppo, Simona Massa

**Affiliations:** ^1^ Interventional Pulmonology Unit V Monaldi Hospital Naples Italy; ^2^ Department of Thoracic Surgery V Monaldi Hospital Naples Italy; ^3^ Pulmonology Unit AO Santobono Hospital Naples Italy; ^4^ Emergency Department C.T.O. Hospital Naples Italy; ^5^ Complex Operative Unit of Pathology Monaldi Hospital Naples Italy

**Keywords:** Carcinoid tumour, endobronchial treatment, hamartoma, lung lobectomy

## Abstract

We describe a patient with incidental endobronchial synchronous hamartoma and typical carcinoid with different management strategy.

## Graphical abstract

We describe a patient with incidental endobronchial synchronous hamartoma and typical carcinoid and the management strategy.

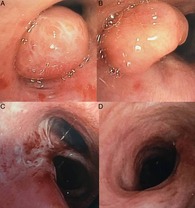

## Clinical Image

**Figure 1:**
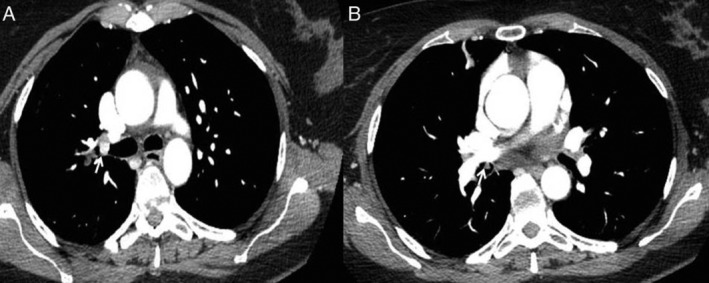
Contrast‐enhanced chest computed tomography (CT) showed a vascularized round lesion with sub‐occlusion of the upper right bronchus (A) and a triangular lesion on intermedius bronchus (B).

**Figure 2:**
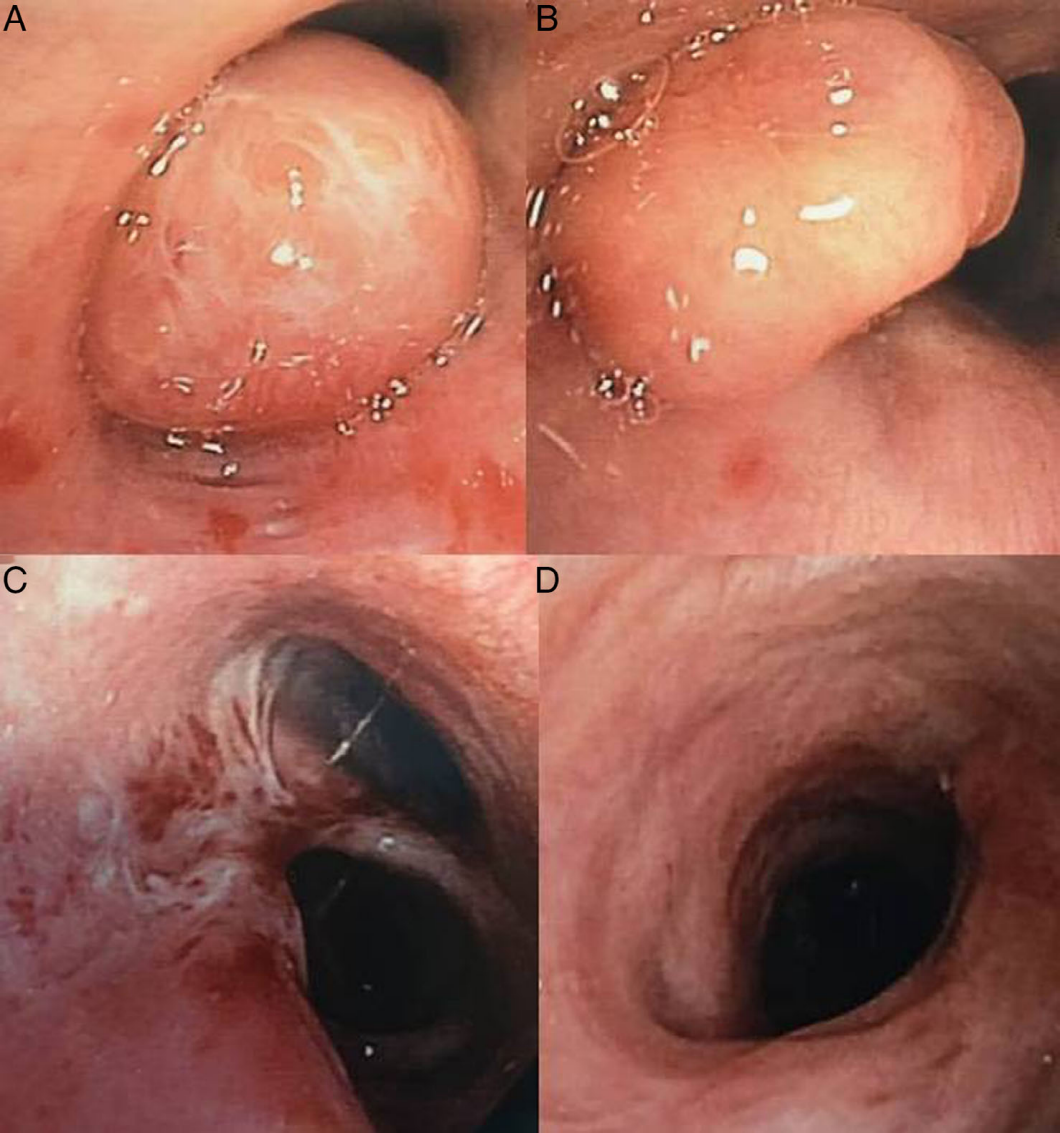
Bronchoscopy view revealed a round‐shaped vascularized lesion sub‐obstructing the right upper lobe bronchus (A) and soft polypoid lesion originating from the distal bronchus intermedius (B). Bronchial scarring post laser‐assisted mechanical resection (C) and closure of bronchial stump following upper right lobectomy (D).

**Figure 3:**
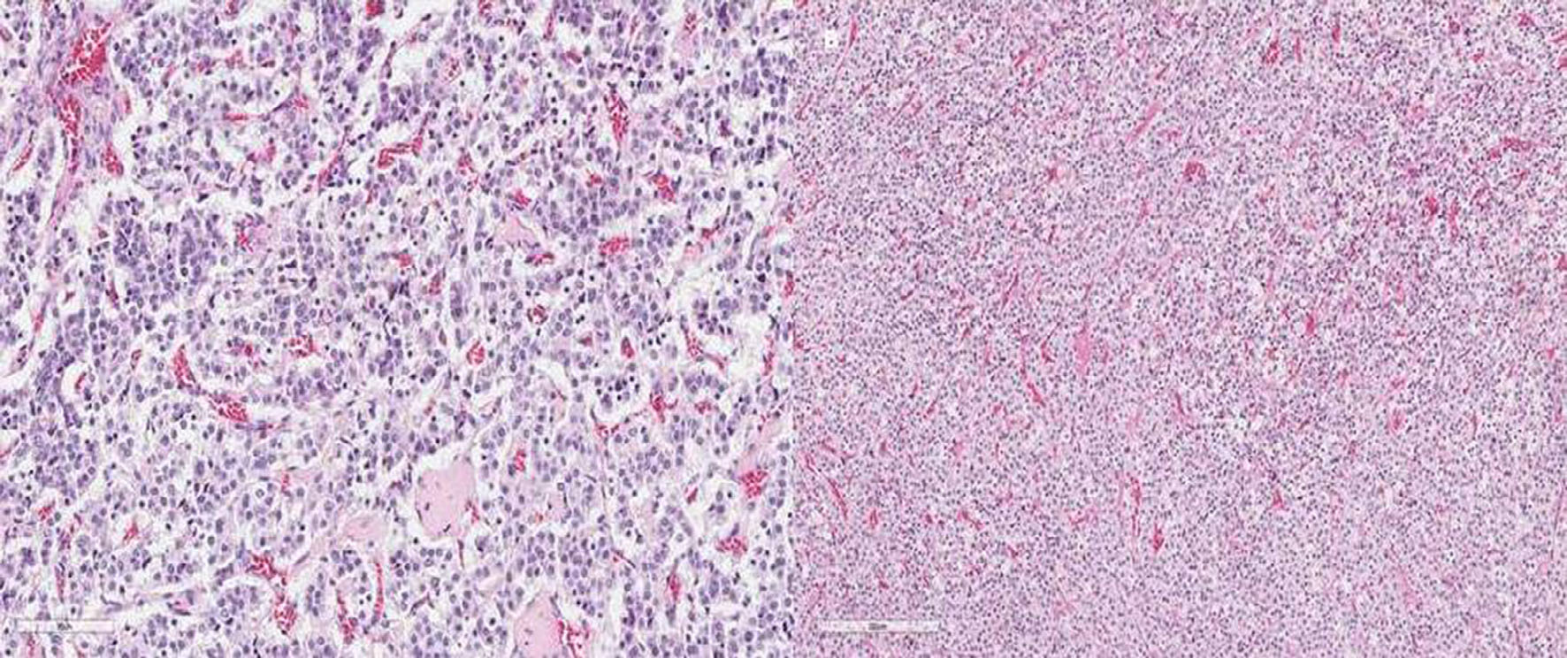
Moderately cellular cartilage fragments without atypia. (A) The polygonal tumour cells arranged in organoid and trabecular growth pattern (haematoxylin–eosin (H–E) stain, 2 mm). (B) Abundant eosinophilic cytoplasm with round‐oval nuclei and inconspicuous nucleoli were observed (H–E stain, 10×).

A 49‐year‐old woman was admitted to our hospital due to paroxysmal dry cough and mild dyspnoea. She was treated with inhaled steroid and bronchodilator for asthma. On contrast‐enhanced chest computed tomography (CT) scan, a solid endobronchial lesion totally occluding the right upper bronchus and an incidental triangular‐shaped lesion on intermedius bronchus were shown (Fig. 1). Fibrobronchoscopy demonstrated a round‐shaped vascularized lesion with typical carcinoid findings located at the orifice of the right upper lobe bronchus and a soft polypoid lesion originating from the medial wall of the bronchus intermedius (Fig. 2A, B). Histological examinations revealed typical carcinoid and hamartoma. A different management strategy was performed [1]. Rigid bronchoscopy treatment with yttrium aluminium garnet (YAG) laser and right upper lobectomy allowed complete removal of the hamartoma and typical carcinoid [1, 2](Fig. 3).

### Disclosure Statement

Appropriate written informed consent was obtained for publication of this case report and accompanying images.
